# Scalable and Robust Regression Methods for Phenome-Wide Association Analysis on Large-Scale Biobank Data

**DOI:** 10.3389/fgene.2021.682638

**Published:** 2021-06-15

**Authors:** Wenjian Bi, Seunggeun Lee

**Affiliations:** ^1^Department of Medical Genetics, School of Basic Medical Sciences, Peking University, Beijing, China; ^2^Department of Biostatistics, University of Michigan, Ann Arbor, MI, United States; ^3^Center for Statistical Genetics, University of Michigan, Ann Arbor, MI, United States; ^4^Graduate School of Data Science, Seoul National University, Seoul, South Korea

**Keywords:** phenome-wide association studies, electronic health records-EHR, saddlepoint approximation, biobank data analysis, unbalanced phenotypic distribution, genetic relatedness, mixed model approaches

## Abstract

With the advances in genotyping technologies and electronic health records (EHRs), large biobanks have been great resources to identify novel genetic associations and gene-environment interactions on a genome-wide and even a phenome-wide scale. To date, several phenome-wide association studies (PheWAS) have been performed on biobank data, which provides comprehensive insights into many aspects of human genetics and biology. Although inspiring, PheWAS on large-scale biobank data encounters new challenges including computational burden, unbalanced phenotypic distribution, and genetic relationship. In this paper, we first discuss these new challenges and their potential impact on data analysis. Then, we summarize approaches that are scalable and robust in GWAS and PheWAS. This review can serve as a practical guide for geneticists, epidemiologists, and other medical researchers to identify genetic variations associated with health-related phenotypes in large-scale biobank data analysis. Meanwhile, it can also help statisticians to gain a comprehensive and up-to-date understanding of the current technical tool development.

## Introduction

With the advances in genotyping technologies and electronic health records (EHRs), large biobanks genotype and extensively phenotype hundreds of thousands of individuals (Greely, 2007; Häyrinen et al., 2008; De Souza and Greenspan, 2013; Nielsen et al., 2018; Wolford et al., 2018; Beesley et al., 2019). For example, UK Biobank is a national and international health resource that collected whole-genome scale genetic data, thousands of complex traits and exposures from ICD billing codes, web surveys, and lab measurements on ∼500,000 individuals (Fry et al., 2017; Bycroft et al., 2018; Canela-Xandri et al., 2018). Other population-based biobanks include All of Us (All of Us Research Program Investigators., 2019), Biobank Japan (BBJ; Nagai et al., 2017), China Kadoorie Biobank (Chen et al., 2011), Nord-Trøndelag Health Study (HUNT) (Krokstad et al., 2013) et al. These datasets can be great resources to identify and validate genetic associations on a genome-wide and even a phenome-wide scale.

Phenome-wide association studies (PheWAS) utilize large numbers of measured phenotypes and can explore the associations between one genetic variant and the entire phenome (Denny et al., 2010). Benefit from the large sample size and extensive traits in analysis, PheWAS in biobanks have the potential to discover novel associations for translational and clinical research, including to construct risk prediction models for complex diseases and phenotypes (Fritsche et al., 2018; Torkamani et al., 2018), to identify the causal effect of exposures and drugs (Verbanck et al., 2018), and to identify drug targets and repurposing (Lam et al., 2017; Pushpakom et al., 2019).

To date, several PheWAS have been performed on biobank data (Roden et al., 2010; Bush et al., 2016). More than thousands of phenotypes have been analyzed at the variant level (Elliott et al., 2018; Zhou et al., 2018; Jiang et al., 2019), gene level (Zhao et al., 2020; Zhou et al., 2020), and pathway level (Dutta et al., 2021). Recently, web-based tools, such as PheWeb (Gagliano Taliun et al., 2020), were developed for visualizing, navigating and sharing the analysis results. All these efforts enable us to provide important insights into many aspects of human genetics and biology. However, due to huge computational burden, unbalanced phenotypic distribution, and genetic relatedness among individuals, PheWAS on large-scale biobank data urgently require more efficient and accurate algorithms.

In this paper, we review challenges in biobank data analysis and regression approaches to addressing these challenges with the goal of providing a practical guidance to statisticians, epidemiologists, and other medical researchers. In section “Statistical and Computational Challenges in Biobank Data Analysis and Approaches to Addressing Them,” we discuss statistical and computational challenges of genome-wide association studies (GWAS) and PheWAS on large-scale biobank data. In section “Scalable and Robust Association Testing Methods,” we summarize recently developed scalable and robust regression approaches. In section “Phenome-Wide Biobank Data Analysis Results and Phewebs,” we introduce existing phenome-wide analyses results. In section “Future Challenges,” we mention potential future challenges which require more advanced methods and tools.

## Statistical and Computational Challenges in Biobank Data Analysis and Approaches to Addressing Them

In this section, we give a brief discussion about statistical and computational challenges in large-scale biobank data analysis and useful strategies to address these challenges (see Table 1).

**TABLE 1:** Statistical and computational techniques on large-scale biobank data analysis.

	Advantage	Disadvantage
**Large computational burden**		
Wald test and likelihood ratio test	can provide accurate estimation of effect size, e.g., odds ratio	is slow to fit large numbers of full models
Score test	does not require fitting full models, and thus is fast when testing marginal genetic effects	cannot provide accurate estimation of effect size; is slow when testing marginal G × E effects
Score test with matrix projection	only requires fitting a covariates-only model, and thus is fast when testing marginal G × E effects	is less accurate when the marginal genetic effect is large
**Unbalanced phenotypic distribution**		
Normal distribution approximation	is fast; is accurate when the phenotypic distribution is balanced, or test statistics are close to the mean value	is not accurate if the phenotypic distribution is unbalanced and the test statistics are far away from the mean value
Firth penalized likelihood-ratio test	is accurate in terms of effect size estimation and testing	is slow for exact Firth test; cannot be used with score test and random effect model
Saddlepoint approximation (SPA) and empirical SPA	uses the entire CGF, and thus is very accurate; is still fast through a hybrid strategy; empirical SPA does not need a closed-form expression of CGF	cannot be used to estimate the effect size
**Genetic relatedness among individuals and mixed models**		
Preconditional conjugate gradient (PCG) with full GRM	does not need storing full GRM, and thus can reduce the memory usage; can easily apply parallel computation; LOCO can avoid proximal contamination due to LD	is slow when sample size and the number of the variants to construct full GRM are very large; LOCO takes more computation time and memory usage.
Sparse GRM	is very fast and requires less memory usage	can be less powerful than using full GRM since the sparse GRM cannot incorporate polygenic effects
Penalized approaches (Regenie)	is fast and requires less memory usage	lacks statistical support to validate its accuracy
		

### Computational Burden, Score Test, and Matrix Projection

**FIGURE 1:**
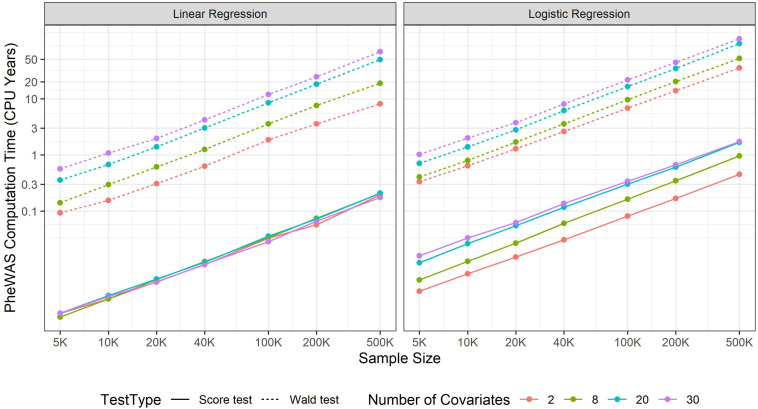
PheWAS computation time. The computation time is evaluated at CPU core of Intel i7-7700T 2.90GHz and then projected to a phenome-wide association studies including 100 balanced binary phenotypes and 10 million variants.

Increasing sample size contributes to more statistical power to identify novel marginal genetic effects and gene-environment interaction (G × E) effects. Meanwhile, it also results in a larger computational burden, which should be carefully handled. In GWAS, most regression approaches include covariates such as age, sex, and top SNP-derived principal components (PCs) to adjust for. Wald and likelihood ratio tests require fitting full models and thus both genetic and covariate effects are simultaneously estimated for all variants. If the number of covariates is large, it will take a substantial amount of time. For example, suppose that 20 covariates were adjusted to fit a standard logistic model, as the sample size increases from 5,000 to 500,000, the computation time increases from 0.02 to 2.55 s. If projected to a PheWAS with 10 million genetic variants and 100 phenotypes, the corresponding computation time increases from 238.3 CPU days to 80.8 CPU years (see Figure 1). Hence, in a large-scale PheWAS, it is not practical to use Wald and likelihood ratio tests even if multiple CPU cores are used for parallel computation.

**FIGURE 2:**
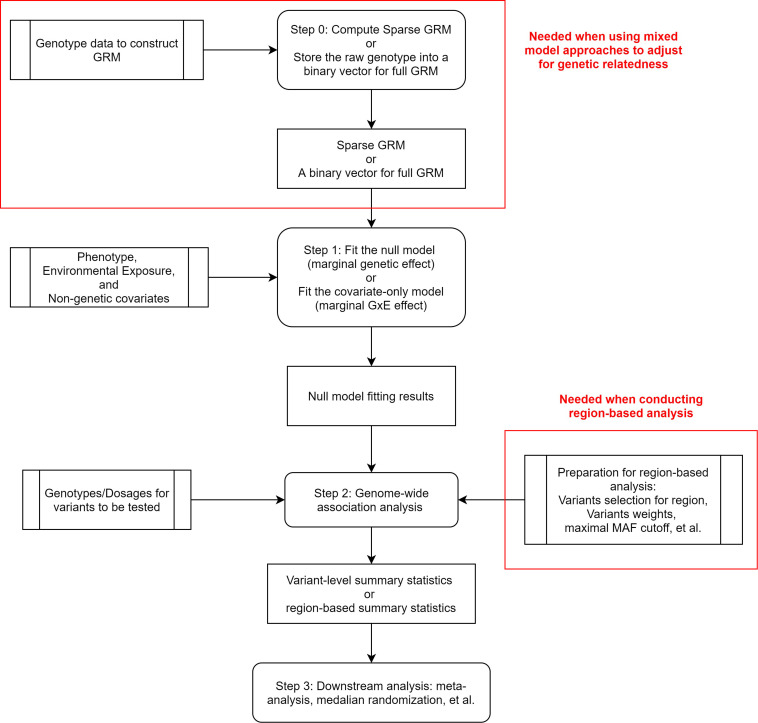
Flowchart of score test association analysis.

In contrast to Wald and likelihood ratio tests, score test does not require fitting the full model. Score test contains two steps: (1) fitting a model under the null hypothesis; (2) calculating score statistics and *p* values for each variant (see Figure 2). When testing marginal genetic effects, the null hypotheses for all variants are the same. Hence, across a genome-wide analysis, score test only requires fitting one null model, which greatly reduces the computation time. Recently, many scalable methods based on score test have been developed to analyze quantitative traits (Zhou and Stephens, 2012; Loh et al., 2015; Jiang et al., 2019), binary traits (Zhou et al., 2018, 2020), time-to-event data (Bi et al., 2020; Dey et al., 2020; He and Kulminski, 2020), and ordinal categorical data (Bi et al., 2021).

Score test is successful in reducing computation time since it avoids duplicated computation to adjust for covariates. This strategy can also be applied to other cases. Recently, scalable methods were proposed for a genome-wide G × E analysis (Bi et al., 2019; Wang et al., 2020). When testing G × E effect, the null model should include marginal genetic effect. Hence, different genetic variants correspond to different null models and the regular score test is not scalable in a large-scale biobank data analysis. Instead of fitting a null model including both covariate and marginal genetic effects, the new methods fit a covariates-only model in Step 1 and then use matrix projection to adjust for the marginal genetic effect in Step 2. Because only one covariates-only model fitting is required for a genome-wide analysis, this strategy can greatly reduce the computation time. However, the matrix projection approach might be inaccurate if the marginal genetic effect is large. To balance the computational efficiency and accuracy, SPAGE (Bi et al., 2019) uses a hybrid strategy as follows. If the marginal genetic effect is small or moderate (e.g., *p* value > 5e-3), the matrix projection is used. Otherwise, regular approaches are used to test the marginal G × E effect.

### Unbalanced Phenotypic Distribution, Firth Bias Correction, and Saddlepoint Approximation

For most of the population-based biobanks, individuals are recruited following a cohort study design, that is, a representative sub-population of the source population are recruited (Beesley et al., 2019). For example, UK Biobank invited all residents aged 40–69 who lived within 25 miles of one of their 22 assessment centers to participate (Bycroft et al., 2018). Due to no stratified sampling, the proportion of rare conditions in biobanks could be very low. For example, in the UK Biobank data, most binary phenotypes based on PheCodes (1,431 out of 1,688; 84.8%) have a case-control ratio lower than 1:100 (Zhou et al., 2018). The unbalanced phenotypic distribution would cause an inflation of type I error rates.

Based on a penalized likelihood function, the Firth’s approach can correct the first-order asymptotic bias of parameter estimates (Firth, 1993). Firth bias correction likelihood ratio test is well calibrated and robust for testing low frequency and rare variants in unbalanced studies (Ma et al., 2013). However, the exact Firth’s method still lacks computational efficiency because it involves fitting the full model (Dey et al., 2017). Recently, Rounak et al. proposed a fast genotype odds ratios estimation in which Firth’s penalty was adjusted (Dey and Lee, 2019). REGENIE also used an approximate Firth regression in which covariate effects were incorporated through an offset term (Mbatchou et al., 2021). These strategies reduce the number of predictors and thus are scalable in GWAS.

If the phenotypic distribution is unbalanced, the underlying null distribution of score test statistics could be highly skewed. Thus, regular normal distribution approximation often fails since only the first two cumulants (i.e., mean and variance) are used (Dey et al., 2017). As an alternative approach, saddlepoint approximation (SPA) uses the entire cumulant-generating function (CGF) to estimate the null distribution, which considerably improve type I error rate control (Daniels, 1954; Jensen, 1995). Recently, SPA is attracting more attention in GWAS and PheWAS (Dey et al., 2017; Zhou et al., 2018, 2020; Bi et al., 2019; Zhao et al., 2020). Extensive simulation studies and real data analysis suggest that SPA greatly outperforms the regular methods especially when testing low-frequency variants. Although more accurate, SPA takes more time than the regular normal distribution approximation. Using the fact that many elements of the genotypes are zeroes (i.e., homozygous major genotypes), the computation of SPA can be speeded up through a partial normal approximation (Dey et al., 2017; Bi et al., 2021). Another strategy is to use SPA only if the normalized score statistics is far away from 0 (e.g., >2), and to use the regular normal distribution approximation otherwise (Dey et al., 2017; Bi et al., 2020).

For SPA, one important step is to estimate CGF of the score test statistic, *S*, under the null hypothesis, i.e., *K*(*t*) = log(*E*_*H*_0__(*e*^*tS*^)). When analyzing binary or ordinal categorical traits, the score test statistic is the sum of multiple random variables, each of which follows a Bernoulli distribution (Dey et al., 2017; Bi et al., 2019, 2021). Hence, the CGF can be explicitly expressed. However, in certain cases, the CGF cannot be expressed in a closed form, which limits the use of SPA. Recently, an empirical SPA approach was used to estimate the CGF (Bi et al., 2020). This approach has been successfully applied to time-to-event data analysis. Since the empirical SPA approach does not rely on the theoretical expression of CGF, it can be used to analyze other complex traits.

### Genetic Relatedness and Mixed Model

As sample sizes continue to increase, many biobanks contain a large proportion of individuals with genetic relatedness. For example, in Nord Trøndelag Health Study (HUNT) (Krokstad et al., 2013), around 81% of the individuals have at least a third degree relative that is also in the study. If not carefully addressed, the genetic relatedness can inflate type I error rates. Including SNP-derived PCs as covariates can relieve it to a certain degree but is not accurate enough. During the past decade, efficient mixed model approaches have emerged as promising solutions (Kang et al., 2010; Zhou and Stephens, 2012; Loh et al., 2015; Zhou et al., 2018; Bi et al., 2021).

Using genome-wide genetic data, the genetic relatedness among individuals can be characterized by a genetic relationship matrix (GRM) (Astle and Balding, 2009; Aguilar et al., 2011). The off-diagonal elements in GRM are close to the kinship coefficients between two individuals. In addition to the fixed effects of covariates, a random effect is included to account for the genetic relatedness. The random effect is assumed following a multivariate normal distribution with a covariance matrix of GRM. For mixed model approaches, the SNP-derived PCs can also be included as covariates to better adjust population stratification (Zhang and Pan, 2015). Mixed model approaches have been proposed to analyze quantitative trait (Kang et al., 2010; Zhou and Stephens, 2012; Loh et al., 2015), binary trait (Zhou et al., 2018, 2020), time-to-event data (Dey et al., 2020; He and Kulminski, 2020), and ordinal categorical data (Bi et al., 2021).

It is technically challenging to apply mixed model approaches in large-scale data. For example, the memory storage of the GRM is O(*n*^2^), where *n* is the sample size. Suppose that sample size *n* = 408, 961 (white British participants in UK Biobank), then it takes 669 Gb of memory to store the GRM given a float-precision format. Instead of precomputing a GRM and then storing it into the memory, an alternative approach is to store the raw genotype (used to construct GRM) into a bitwise binary vector and then load it when in usage (Loh et al., 2015). Suppose that *m* = 93,511 variants are used to calculate GRM, the memory usage can be reduced to 9.56 GB. Another computational challenge is to fit the model under the null hypothesis which requires either performing spectral decomposition on GRM or calculating the inverse of the *n* × *n* matrices, both require O(*n*^3^) calculation. Instead, a linear system solver, such as the preconditional conjugate gradient (PCG) approach can be used to provide scalable computation, which requires O(*mn*^1.5^) (Kaasschieter, 1988). PCG is easily parallelizable, so parallel computing libraries, such as OpenMP (Dagum and Menon, 1998) and RcppParallel (Allaire et al., 2018), can be used to fully utilize the available CPU cores as many as possible.

Although scalable to analyze hundreds of thousands of individuals, the mixed model approaches using full (dense) GRM are still computationally intensive. A straightforward approach is to use a sparse GRM in which values less than a pre-given cutoff (e.g., <0.05) were set to 0 (Jiang et al., 2019; Bi et al., 2021). This approach can substantially reduce computation time and memory usage. However, using sparse GRM can be less powerful than using full GRM since the sparse GRM cannot incorporate polygenic effects (Jiang et al., 2019).

When analyzing a candidate variant, the variants in linkage disequilibrium with it (including the candidate variant itself) should not be used to construct GRM to avoid modeling effects twice (Yang et al., 2014). To avoid the proximal contamination, leave one chromosome out (LOCO) scheme is used in linear mixed model approaches (Lippert et al., 2011; Yang et al., 2014; Loh et al., 2015). For binary trait, sensitivity analyses suggested that the proximal contaminations in GWAS for diseases with low prevalence is not as substantial as for more common diseases and thus LOCO scheme might not be required (Zhou et al., 2018).

Instead of the mixed effect model framework, a fixed effect model with a penalty can be used to account for genetic relatedness. A recent developed REGENIE (Mbatchou et al., 2021) used two-step ridge regressions to calculate predictors from genetic data and then used linear and logistic regression to associate quantitative and binary traits with genetic variants. Compared to mixed effect model approaches, fixed effect model approaches can be faster and needs a smaller amount of memory. Although shown to perform similarly as BOLT-LMM (Loh et al., 2015) and SAIGE (Zhou et al., 2018) when applying to UK Biobank, it is not clear whether the genetic relatedness can be well characterized by the fixed effect model if the participants are in a highly related or in a multiethnic cohort study.

## Scalable and Robust Association Testing Methods

In this section, we introduce regression methods that are scalable and robust in large-scale biobank data analysis (see Table 2). We let *G* denote the genotype of genetic variant and *X* denote the confounding covariates. The corresponding coefficients are β_*G*_ and β_*X*_, respectively. For mixed models, we let Φ denote GRM, σ denote variance component, *b* denote the random effect and assume that *b* follows a multivariate normal distribution *N*(0,σΦ). The trait of interest is denoted as *Y*.

**TABLE 2:** Summary of analysis methods and software.

		Accounts for unbalanced phenotypic distribution	Accounts for sample relatedness	Software website
Variant-level analysis				
BOLT-LMM (Loh et al., 2015)	Quantitative trait	N/A	Support full GRM	https://alkesgroup.broadinstitute.org/BOLT-LMM
fastSPA (Dey et al., 2017)	Binary trait	Support SPA	NO	https://cran.r-project.org/web/packages/SPAtest
SAIGE (Zhou et al., 2018)	Binary and quantitative trait	Support SPA	Support full GRM	https://github.com/weizhouUMICH/SAIGE
fastGWA (Jiang et al., 2019)	Quantitative trait	N/A	Support sparse GRM	https://cnsgenomics.com/software/gcta/#fastGWA
REGENIE (Mbatchou et al., 2021)	Binary and quantitative trait	Support SPA and Firth bias correction methods	Use panelized approaches	https://github.com/rgcgithub/regenie
SPAGE (Bi et al., 2019)	G × E analysis for binary trait	Support SPA	NO	https://github.com/WenjianBI/SPAGE
SPACox (Bi et al., 2020)	Time-to-event data	Support SPA	NO	https://github.com/WenjianBI/SPACox
COXMEG (He and Kulminski, 2020)	Time-to-event data	NO	Support sparse GRM	https://cran.r-project.org/web/packages/coxmeg/
GATE (Dey et al., 2020)	Time-to-event data	Support SPA	Support both sparse and full GRMs	https://github.com/weizhou0/GATE
POLMM (Bi et al., 2021)	Ordinal categorical data	Support SPA	Support both sparse and full GRMs	https://github.com/WenjianBI/POLMM
**Region-level analysis**				
Robust SKAT (Zhao et al., 2020)	Binary trait	Support SPA	NO	https://cran.r-project.org/web/packages/SKAT
SAIGE-Gene (Zhou et al., 2020)	Binary and quantitative trait	Support SPA	Support full GRM	https://github.com/weizhouUMICH/SAIGE
MAGEE (Wang et al., 2020)	G × E analysis	NO	Support full GRM*	https://github.com/xwang21/magee
STAAR (Li et al., 2020)	Binary and quantitative trait	NO	Support sparse GRM	https://github.com/xihaoli/STAAR

*Full GRM is pre-calculated and stored, which could take large amount of memory and might be not applicable when sample size is very large.

### Quantitative Traits

Linear regression is the most widely used approach when the trait of interest is measured quantitatively. When analyzing unrelated individuals, the regular linear model is

$$Y=\beta_{X}X+\beta_{G}G+\epsilon$$

where ϵ is an error term that is usually assumed normally and independently distributed. The test of null hypothesis *H*_0_:β_*G*_ = 0 is implemented in several tools including GCTA (Yang et al., 2011), plink (Chang et al., 2015), et al. To adjust for the genetic relatedness, additional random effect *b* should be included and the LMM (Kang et al., 2010; Zhou and Stephens, 2012) is

$$Y=\beta_{X}X+\beta_{G}G+b+\epsilon .$$

BOLT-LMM (Loh et al., 2015) proposed to compactly store the genotype used to construct a full GRM Φ in memory and applied PCG to efficiently fit the null mixed model. fastGWA (Jiang et al., 2019) used sparse GRM to further reduce the computation time and memory usage. If the distribution of quantitative trait is highly skewed, inverse-normal transformation is commonly used to convert the raw quantitative trait.

### Binary Traits

For complex disease research, individuals are usually divided into two groups: the cases (*Y* = 1) or the controls (*Y* = 0). Logistic model and logistic mixed model can model the dependence of a binary trait on covariates and genetic variants as below.

$$\begin{array}{c} \log\frac{\mathrm{Pr}\left(Y=1\right)}{1-\mathrm{Pr}\left(Y=1\right)}=\beta_{X}X+\beta_{G}G,\\ \log\frac{\mathrm{Pr}\left(Y=1\right)}{1-\mathrm{Pr}\left(Y=1\right)}=\beta_{X}X+\beta_{G}G+b. \end{array}$$

In the presence of population stratification, applying LMMs to binary traits can lead to incorrect type I error rates, particularly when the population groups have heterogeneous case-control ratios (Chen et al., 2016). This is because LMM assumes that the variance of the binary trait is constant and does not change with the mean (Jarque and Bera, 1980). Based on the logistic mixed model, Chen et al. (2016) developed a score test called GMMAT. GMMAT uses penalized quasi-likelihood (Breslow and Clayton, 1993) and average information restricted maximum likelihood algorithm (Gilmour et al., 1995) to fit the null mixed model. However, GMMAT package requires storing a precalculated GRM, which takes huge amount of memory when sample size is very large (e.g., >100,000). In addition, it cannot control type I error rates when case-control ratio is unbalanced. To address these challenges, SAIGE method applied computational strategies as in BOLT-LMM to avoid storing GRM and used SPA for testing (Zhou et al., 2018). Recently, more optimized tools have been developed to increase the computational performance of SAIGE (Zheng and Davis, 2020).

### Ordinal Categorical Traits

Ordinal categorical trait is an extension of binary trait to measure more conditions. It is widely used in surveys, questionnaires, and tests to measure human behaviors, satisfaction, and preferences (Agresti, 2003). For example, hedonic scale of liking ranging from “extremely dislike” to “extremely like” was widely used to measure preferences (Bi et al., 2021). Although usually coded as numeric values, the ordinal categorical data is different from quantitative trait since the values cannot characterize the underlying scale well (Agresti, 2003).

Suppose that *Y* = 1, 2, *J* is to denote an ordinal categorical phenotype with *J* ordinal conditions. Recently, proportional odds logistic mixed model (POLMM) (Bi et al., 2021) has been used to model the ordinal categorical phenotype as follows

$$\text{logit}\left(\nu_{j}\right)=\epsilon_{j}-\beta_{X}X-\beta_{G}G-b,1\leq j\leq J$$

where ν_*j*_ = Pr(*Y* ≤ *j*|*X, G, b*) is the cumulative probability of the phenotype *Y* ≤ *j* and ϵ: ϵ_1_ < … < ϵ_*J*_ = ∞ were used to categorize the data. POLMM supports both full (dense) GRM and sparse GRM when fitting the null model and is scalable to analyze biobanks with hundreds of thousands of individuals. In addition, POLMM uses SPA and thus is robust when testing low-frequency and rare variance even if the phenotypic distribution is highly unbalanced (Bi et al., 2021).

### Time-to-Event Data

Time-to-event data is unique because the outcome of interest is not only whether an event occurred, but also when the event occurred (Altman and Bland, 1998). In medical studies, time-to-event data were often used to characterize outcomes such as death and cancer progression (Tolles and Lewis, 2016). Another unique feature of the time-to-event data is censoring, that is, not all subjects experience the event by the end of the follow-up period. With the increasing use of EHRs and biobanks for genetics research, time-to-event data analysis is becoming more common in genetic studies of human diseases (Huang et al., 2009; Kapoor et al., 2014).

Cox proportional hazard (PH) model is widely used to analyze time-to-event data (Cox, 1972). The Cox PH model specifies the hazard function λ(*t*) for the failure time associated with genotype and covariates as below.

$$\lambda \left(t\right)=\lambda_{0}\left(t\right)\exp\left(\beta_{X}X+\beta_{G}G\right)$$

where λ_0_(*t*) is a baseline hazard function. R package gwasurvivr (Rizvi et al., 2018) was developed to perform genome-wide survival analysis. To increase the computational efficiency, gwasurvivr first fits null model with β_*G*_ = 0 and then uses the parameter estimates as initial points when testing variants. Since gwasurvivr is based on a Wald test, it is still not scalable when the sample size is large (>100,000). Recently, a fast and accurate method called SPACox was proposed to use an empirical SPA to calibrate *p* values (Bi et al., 2020). SPACox is based on a score test and is more robust to analyze low-frequency and rare variants, especially when the event rate is moderate or low.

If random effect is included to adjust for genetic relatedness, the corresponding mixed model (i.e., frailty model) is

$$\lambda \left(t\right)=\lambda_{0}\left(t\right)\exp\left(\beta_{X}X+\beta_{G}G+b\right).$$

Existing methods such as COXMEG (He and Kulminski, 2020) and GATE (Dey et al., 2020) are scalable in large-scale GWAS. When fitting the null model, COXMEG supports sparse GRM and GATE supports both full and sparse GRM. In addition, GATE uses SPA to calibrate *p* values, which makes it more powerful and robust to analyze low-frequency and rare variants.

### Gene-Environment Interaction Analysis

Gene-environment interaction (G × E) plays an important role in the etiology of many complex traits (Gauderman et al., 2017; McAllister et al., 2017). For a binary trait *Y*, the full logistic model for G × E is as follows.

$$\log\frac{\mathrm{Pr}\left(Y=1\right)}{1-\mathrm{Pr}\left(Y=1\right)}=\beta_{X}X+\beta_{E}E+\beta_{G}G+\beta_{G\times E}\cdot \left(G\times E\right)$$

where *E* is environmental factor and *G* × *E* is the interaction term. One strategy to reduce computation time is to test both marginal and interaction effects of *G*: β_*G*_ = β_*G* × *E*_ = 0, which share the same null hypothesis in the entire genome and hence can greatly reduce computation time if the score test is used. However, since the marginal genetic effect is usually larger than the G × E effect, the association identified by the joint test is mostly driven by the marginal genetic effect, which is not the major interest in a G × E study.

Software packages such as CGEN (Bhattacharjee et al., 2010) and GxEScan (Gauderman et al., 2013) have been developed for a genome-wide G × E analysis. Since these tools mainly implement the Wald test, the computation burden is still very high in a large-scale biobank data analysis. To improve the efficiency, several two-step approaches have been proposed (Kooperberg and LeBlanc, 2008; Murcray et al., 2008). These methods compute screening *p* values to test marginal genetic associations (i.e., β_*G*_ = 0) or the dependency between *E* and *G*. Then, the variants with significant screening *p* values are selected to test G × E effect in the next step. In addition to the single-variant tests, various set-based methods have been proposed to test G × E effect (Chen et al., 2014; Lin et al., 2016; He et al., 2017b; Su et al., 2017). These methods jointly test variants in a particular gene or functional region to increase power for low-frequency and rare variants.

Recently, more efficient approaches have been proposed for genome-wide G × E analysis (Bi et al., 2019; Wang et al., 2020). Instead of fitting a null model, these methods fit a covariates-only model and then use matrix projection to calculate score statistics and *p* values. SPAGE (Bi et al., 2019) uses SPA to calibrate *p* values and thus is more robust even if the case-control ratio is unbalanced. MAGEE (Wang et al., 2020) is a set-based method which is developed based on mixed model and can identify associations between an aggregate variant set and environmental exposures on quantitative and binary traits.

### Region-Based Rare Variant Test

When testing for low-frequency and rare variants, the statistical power of single-variant based association tests is usually low. Region-based approaches can boost power by evaluating association for multiple variants in a biologically relevant region, such as gene (Wu et al., 2011; Lee et al., 2012a,b, 2013, 2014; Ionita-Laza et al., 2013). Burden tests collapse rare variants into genetic scores and are powerful when a large proportion of variants are causal and the effects are in the same direction (Morgenthaler and Thilly, 2007; Li and Leal, 2008). Variance-component testing approaches, such as SKAT, test variance of genetic effects and are more powerful in the presence of variants with different effect directions or a small fraction of causal variants (Pan, 2009; Neale et al., 2011; Wu et al., 2011). Combined tests, such as SKAT-O (Lee et al., 2012a,b) and ACAT (Liu et al., 2019) methods, can combine burden and variance-component tests and are more robust in different scenarios.

R package SKAT is a useful generic tool for region-based rare variant analysis. Besides the original Burden, SKAT, and SKAT-O methods, features including efficient resampling (ER) (Lee et al., 2016), combined test of common and rare variants (Ionita-Laza et al., 2013), and X chromosome test (Ma et al., 2015) are also supported. To control for unbalanced case-control ratio, Zhao et al. proposed robust region-based association approaches. The robust approaches use SPA and ER to calibrate *p* values and can control type I error rates when the case-control ratio is unbalanced (Zhao et al., 2020). SMMAT is an extension of GMMAT into the region-based association analysis (Chen et al., 2019). SMMAT can adjust for genetic relatedness but is not applicable when the sample size is large or the case-control ratio is unbalanced. SAIGE-Gene (Zhou et al., 2020) can incorporate a full GRM to account for genetic relatedness and is scalable and accurate to analyze hundreds of thousands of individuals. Recently, integrative region-based association approaches were proposed to incorporate multiple functional annotations of genetic variation (He et al., 2017a; Li et al., 2020). If the variant risk status can be predicted by functional annotations, these approaches can significantly improve power.

## Phenome-Wide Biobank Data Analysis Results and Phewebs

In this section, we highlight existing phenome-wide analyses results. The usage of PheWeb facilitates the sharing and organizing of genetic association results.

• Oxford Brain Imaging Genetics (BIG) Server version 2.0 can browse GWAS results for UK Biobank Brain Imaging Phenotypes and other traits/diseases. The primary source included results from 3,144 GWAS of Brain Imaging Derived Phenotypes (IDPs) measured on 9,707 participants of the UK Biobank study. Currently, the server has loaded more GWAS results including the GWAS results of ∼ 2,000 phenotypes in the UK Biobank processed by Ben Neale^1^.
• The Michigan Genomics Initiative (MGI) is a collaborative research effort among physicians, researchers, and patients at the University of Michigan (U-M). Since most of the PheWAS are based on UK Biobank, the PheWAS data analysis based on MGI is an important supplementary although its sample size (∼40,000) is less than UK Biobank^2^.
• The BBJ project has collected around 200,000 individuals with diseases cases consisting of 47 various diseases. These subjects were recruited from 12 medical institutes in Japan. The analysis results of total 244 phenotypes including both binary and quantitative traits have been released^3^.
• SAIGE method can better control type I error rates when the case-control ratio is unbalanced. Using SAIGE, GWAS on 1403 ICD-based traits were performed based on the White British participants of the UK Biobank^4^.
• POLMM is an extension of SAIGE on ordinal categorical data analysis. PheWAS of 258 ordinal categorical phenotypes on UK Biobank has been conducted, in which 150 phenotypes are to describe food and other health-related preferences^5^.
• Based on the fastGWA method, PheWAS was applied to 2,173 traits on 456,422 array-genotyped as well as 49,960 whole-exome-sequenced individuals of European ancestry in the UK Biobank. Since linear mixed model approaches were used to analyze binary traits and ordinal categorical data, the PheWAS only analyze variants with MAF > 0.01^6^.
• Using the robust SKAT-O approach for binary phenotypes, a total of 18,360 genes were analyzed based on 45,596 independent European samples across 791 binary phenotypes with at least 50 cases. The PheWAS are based on UK Biobank 50K exome data processed by FE pipeline^7^.
• PathWeb displays results for associations between over 10,000 pathways (gene-sets) and phenotypes derived from ICD billing codes of White British participants of the UK Biobank. GWAS summary-statistics obtained using SAIGE for 1,403 binary phenotypes derived from ICD billing codes have been used in the analysis^8^.

## Future Challenges

The recent success in methodology development has greatly facilitated the large-scale biobank data analysis on a genome-wide and phenome-wide scale. In the future, it is expected that more comprehensive information, in terms of both genome and phenome, will be collected and shared through the continuously upgrading biobanks. The rapid development of biobanks provides a basis for precision health and medicine. Meanwhile, it also brings new challenges, which requires more advanced methods and tools. Here we list some of these challenges.

### Larger Sample Sizes

The current biobanks usually recruit half million participants. In the future, we are likely to encounter biobanks with millions of and even tens of millions of participants. For example, All of US biobank in US aims to recruit 1 million individuals and UK announced a plan to recruit 5 million individuals (Scott et al., 2019). The increase of sample size asks for more computational time and memory usage, which should be carefully addressed in terms of methodology and software implement.

### Whole Genome Sequencing

In the coming decades, whole genome sequencing (WGS) will replace GWAS chips and become the most widely used genotyping platform. Since WGS can accurately identify and genotype rare variants, more scalable and powerful strategies and methods to evaluate rare variant associations in whole genome are increasingly needed. The evolving availability of new technologies will provide us with rich multi-omics data resources. Effectively incorporating additional information, such as epigenetics, is also important to boost powers and to increase interpretability in WGS studies.

### Multivariate and High Dimensional Phenotypes

In the past decades, GWAS mainly focus on univariate phenotypes, that is, the phenotype of interest has only one variable. Recently, multivariate and high dimensional phenotypes are increasingly available. For example, longitudinal data track the same sample and collect repeated observations at different time points. Image phenotypes, such as Magnetic Resonance Imaging (MRI) of brain and other organs, are collected to better diagnose and treat diseases. Developing scalable and robust methods to appropriately analyze these complex phenotypes is important to fully utilize these data.

### Effectively Use Large Numbers of Phenotypes

The current phenome-wide analyses are mainly to test single phenotype and then look at the association patterns across phenome. This strategy does not utilize the correlation and causal relationship between phenotypes. Effectively aggregating associations in large numbers of phenotypes can boost statistical powers and gain better phenome-wide understanding. Existing joint tests of multiple phenotypes include MultiPhen (O’Reilly et al., 2012), MANOVA (Stephens, 2013), USAT (Ray et al., 2016), and Multi-SKAT (Dutta et al., 2019). Some of the methods developed for non-human data, such as GPWAS (Liang et al., 2020) for plant genetics, also can be used. In addition, when analyzing multiple phenotypes, the imputation of missing data will be important since removing individuals with at least one missing phenotype will greatly reduce the sample size.

### Multiethnic Studies and Admixed Population

Population structure and family relatedness are major confounders in genetic association studies. The recently proposed mixed effect and fixed effect models are usually applied to individuals from the same ancestry group, as it isn’t clear whether they can accurately analyze multiethnic individuals. Meta-analysis is commonly applied to combine analysis results from different ancestry groups, but it may need more research if individuals of different ancestry groups are related. For admixed population, specialized approach is needed to construct GRM, such as a method using individual specific allele frequency, but scalable mixed model to use this type of GRM is under-developed (Thornton et al., 2012). As biobanks recruit individuals from diverse populations, it would be important to identify and develop optimal methods and tools to analyze multiethnic and admixed individuals.

## Conclusion

The emergence of biobanks allows researchers to explore extensive associations between genetic variants and thousands of complex traits. In this paper, we discussed statistical and computational challenges in large-scale biobank data analysis and reviewed available methods and tools to address these challenges. In addition, we also briefly introduced possible challenges in the future. Benefit from the continuous biobanking efforts to connect genome-wide variants and phenome-wide traits, several PheWAS have been performed and it is expected that more PheWAS results will be available in the coming decade. Scalable and robust statistical approaches will certainly play an essential role in the success. In addition, developing user-friendly software that makes full use of the computing capacity is also important.

## Author Contributions

WB and SL conceived the study and drafted the manuscript. Both authors contributed to the article and approved the submitted version.

## Conflict of Interest

The authors declare that the research was conducted in the absence of any commercial or financial relationships that could be construed as a potential conflict of interest.

## Footnotes

1 http://big.stats.ox.ac.uk/

2 http://pheweb.sph.umich.edu/

3 http://jenger.riken.jp:8080/

4 http://pheweb.sph.umich.edu/SAIGE-UKB/

5 https://polmm.leelabsg.org/

6 http://fastgwa.info/ukbimp/phenotypes

7 https://ukb-50kexome.leelabsg.org/

8 https://ukb-pathway.leelabsg.org/
